# Traumatic Optic Neuropathy: Analysis of Demographic and Clinical Parameters Over Three Years in a Tertiary Care Hospital in India

**DOI:** 10.7759/cureus.31771

**Published:** 2022-11-22

**Authors:** Praveen Kumar K V, Satyasri B, Shashi Ahuja, Praveen Kumar S

**Affiliations:** 1 Ophthalmology, A C Subba Reddy (ACSR) Government Medical College, Nellore, IND; 2 Ophthalmology, Sri Lakshmi Narayana Institute of Medical Sciences, Puducherry, IND; 3 Ophthalmology, Jawaharlal Institute of Postgraduate Medical Education and Research, Puducherry, IND

**Keywords:** ocular injuries, trauma, optic neuropathy, sudden visual loss, head injury

## Abstract

Background

Treatment options for traumatic optic neuropathy (TON) are limited and the role of steroids in the treatment of TON is still controversial. Hence this study was planned to highlight the role of steroids in the treatment of TON.

Purpose

The study aims to highlight the epidemiological and clinical characteristics, as well as the role of steroids, in TON cases seen during a three-year period at a tertiary care center in India.

Methods

This was a retrospective study that reviewed records of all cases of TON between January 2018 to January 2020.

Results

Twenty-three cases of TON were seen representing 1.26% cases of head injuries. The median age was 18 years. One patient (4.34%) had bilateral TON and 18 patients (78.26%) were referred from accident and trauma care. None of the patients presented directly to an ophthalmologist. The most common cause of injury was automobile accident (69.56%). Visual acuity at presentation was 20/80-20/100 in six (26.08%) cases. Fifteen (65.4%) patients had associated closed globe injury. Seven (30.43%) patients had ocular adnexal involvement and 10 (43.47%) patients had orbital fractures. Seventeen (73.91%) received steroid treatment and six of these patients showed visual improvement.

Conclusions

The study showed that there was no significant association between presenting visual acuity and treatment. The presence of significant ocular injury and orbital injury increased the likelihood of treatment. There was no difference between the treated and untreated groups with respect to final visual acuity.

## Introduction

Traumatic optic neuropathy (TON) refers to a direct or an indirect acute injury of the optic nerve secondary to trauma and is a rare cause of severe permanent visual loss. The intracanalicular segment of the optic nerve is the most susceptible to injury because the dural sheath is tightly adherent to the periosteum followed by the intracranial portion of the optic nerve. The reported incidence of TON following blunt trauma varies from 0.7% to 2.5% [1]. A recent national epidemiological survey of TON in the UK found a minimum prevalence in the general population to be one in 1,000,000 [2]. The most common cause of TON in adults has been reported to be road traffic accidents (RTA) (49%), followed by falls (27%) and assaults (13%) [3]. In a pediatric case series, TON was attributed to falls in 50% of the cases and due to RTA in 40% of the cases [4]. The incidence is reported to be 1.8% in head injury patients.

Managing TON remains controversial with treatment regimes that vary from high doses of steroids to surgical decompression. Previously, TON was treated with a megadose steroid regimen, based on the findings from the Second National Acute Spinal Cord Injury Study (NASCIS II) [5]. But the adverse survival effect of megadose steroids in severe head injury identified by the Corticosteroid Randomisation after Severe Head injury (CRASH) study decreased the use of this approach [6]. A review of the literature on TON treatment revealed that 0-48% of TON cases showed spontaneous improvement with no treatment [7,8], 44-82% showed improvement with steroids [9,10] and 35-70% showed improvement with surgical decompression [11-13]. To the best of our knowledge, there are few available published reports that have assessed TON demographics and epidemiological data. Therefore, this study was designed to determine various epidemiological data, clinical characteristics and the role of steroids in TON cases in a tertiary eye care center over a three-year period.

## Materials and methods

This study was a retrospective study. The study was done in a hospital-based setting in a tertiary care center in southern India. Inclusion criteria included all cases of TON treated between January 2018 to January 2020 in the hospital. All cases of trauma where there was an identifiable cause of loss of vision were excluded from the study. The study was registered with the institutional review board and clearance was obtained from the ethics committee of the institute (Jawaharlal Institute of Postgraduate Medical Education and Research, JIPMER). Informed consent was obtained from all the participants in the study.

Medical records of all cases of TON that were referred to the department of Ophthalmology between January 2018 to January 2020 were reviewed. All cases of TON presenting to the department during the study period were included. Cases where the details were not available and patients who did not come for follow-up were excluded from the study. The medical records of the included subjects were obtained from the medical records department and hospital information system of the institute. The diagnosis of TON was entertained in those patients who presented with a history of trauma, particularly head injuries and had sudden onset of diminished vision with a relative afferent pupillary defect, normal fundus and normal neuroimaging. All cases of trauma presenting with signs explaining the loss or diminution of vision were excluded from the study. Details including age, gender, patient referral source, mode of injury, visual acuity at presentation, type of treatment received, number of referrals, investigations done and details of the visual outcome after treatment were collected for all the subjects. The mode of injury was classified as road traffic accident, assault, fall and miscellaneous when it did not fit into the above category. The road traffic accidents were further classified as two-wheeler or four-wheeler accident. The visual acuity at the presentation was recorded using Snellen Visual acuity chart and was classified into four categories: better than 6/18, 6/18-6/60, 6/60 to perception of light and no perception of light. Associated orbital fractures were classified as simple or complex and were recorded. Based on the treatment received the subjects were classified into two groups: one group receiving steroid treatment and other group where no intervention was done. All the collected data was recorded in a Microsoft Access database and analyzed using SPSS software (IBM Corp., Armonk, NY). A Chi-square test and Fisher’s exact test were used to analyze the evidence of association between various variables and a p-value less than 0.05 was considered significant.

## Results

Over a period of three years, 23 cases of TON were reported. The proportion of TON with respect to the number of head injuries over the study period was 1.26%.

Patient demographics and presentation patterns

All cases in our study were males (100%). The median age at the time of the injury was 18 years. Eleven patients (47.82%) were under 19 years of age (Table 1). One patient (4.34%) had a bilateral TON. In the study, 18 patients (78.26%) were referred from accident and trauma care and four patients (17.39%) were referred from the hospital’s neurosurgery department. One patient (4.34%) was referred from an outside public health sector hospital. No patient presented directly to the ophthalmologist (Table 2). All patients were seen by an ophthalmologist on the same day following the injury while one patient that was referred from another location was seen a day after the injury.

**Table 1:** Table showing demographic characteristics of the study patients

CHARACTERISTICS	NUMBER OF PATIENTS	PERCENTAGE
Age		
<19 years	11	47.82
>19 years	12	52.17
Gender		
Male	23	100
Female	0	0
Injured Eye		
Right	14	60.86
Left	9	39.13
Injury Type		
Road Traffic Accident	16	69.5
Assault	2	8.69
Fall	3	13.04
Miscellaneous	2	8.69

**Table 2:** Table showing the clinical characteristics of the study patients ENT: Ear Nose Throat

	Number of patients	Percentage
Ocular Injury		
Anterior Segment	4	17.39
Posterior Segment	4	17.39
Adnexal involvement	7	30.43
Orbital fractures		
Simple	3	13.04
Complex	7	30.43
None	13	56.52
Baseline Visual acuity		
Greater than or equal to 6/18	3	13.04
6/24-6/60	6	26.08
6/60-Perception of light	9	39.13
No perception of light	5	21.73
Source of referral		
Accident and Emergency Department	18	78.26
Neurosurgery department	4	17.39
Referred from outside public sector hospital	1	4.34
Cross Consultations		
Neurosurgery	11	47.82
ENT	8	34.78
Dental	6	26.08
Orthopaedics	4	17.39
Cardiovascular intervention	1	4.34
Medical attention	1	4.34
Treatment		
Steroids	17	73.91
No treatment	6	26.08

Mode of injury

The most common cause of injuries was RTA (69.56%, 16/23) followed by falls (13.04%, 3/23) and assault (8.69%, 2/23). In two patients (8.69%), there were miscellaneous injuries (sports injuries and a combination of falling and assault). Assault was the common mode of injury in individuals under 40 years of age whereas RTA and falls were equally distributed among all other age groups (Table 1). Out of 16 cases of road traffic accidents, 14 (87.5%) were two-wheeler accidents and two (12.5%) were four-wheeler accidents.

Visual acuity at presentation

Visual acuity (VA) was better than 6/18 in three (13.04%) patients, 6/24-6/60 in six (26.08%) patients, 6/60-Perception of light (PL) in nine (39.13%) patients and no PL in five (21.73%) patients (Table 2).

Associated ocular injuries

Out of 23 patients, 15 (65.21%) patients had associated closed globe injury. Four (17.39%) patients had anterior segment involvement which included traumatic uveitis and traumatic mydriasis. Three patients (20%) had associated lid injuries, one patient (6.6%) had conjunctival laceration, two patients (13.3%) had subconjunctival hemorrhage and one patient (6.6%) had corneal involvement in the form of abrasion. Four (17.39%) patients had posterior segment involvement in the form of macular edema, pigment epithelial detachment and optic disc hemorrhage. The patients with fundus involvement were further evaluated by appropriate investigations such as optical coherence tomography (OCT) to confirm TON as the cause of diminished vision (Table 2).

Orbital injuries

Ten (43.47%) patients had orbital fractures in the study; three (13.04%) had simple fractures of the floor, medial and or lateral walls and seven (30.43%) patients had complex fractures of the orbit (Table 2).

Three patients had fracture of the floor of the orbit and seven patients had multiple orbital wall fractures. Multiple fractures appeared to be commonly associated with TON.

Three patients had simple orbital fractures and did not need intervention according to the treating dental surgeon and one patient with floor fracture refused intervention.

Bilateral involvement

One patient in the study had bilateral TON which was due to RTA. The presenting VA was no PL in both eyes and the pupils were dilated and fixed. The patient had skull fractures and intracranial injuries in the form of frontal and parietal lobe contusion, suggesting severe head injury seen usually in bilateral cases of TON. The patient remained PL negative at the six-month follow-up visit.

Radiological investigations

All the patients underwent a brain and orbit CT scan to rule out intracranial and intraorbital lesions (Table 2).

Consultations

The majority of patients in the study had various other injuries for which they were referred to a specialist. Eleven patients (47.82%) had neurosurgical consultations, eight patients (34.78%) were referred to the ENT specialist, six patients (26.08%) were referred to the dental surgeon for orbital fracture repair, four patients (17.39%) were referred to the orthopaedist for limb fracture repair, one patient needed cardiovascular intervention (4.34%) and one patient needed medical attention (4.34%) (Table 2). Cardiovascular intervention was needed as the patient had a pneumothorax and one patient needed medical attention because of hypertension.

Treatment

All patient treatments were reviewed; out of 23 cases, 17 patients (73.91%) received steroid treatment and six patients (26.08%) did not receive acute intervention (Table 2). Intravenous methylprednisolone was given to patients and megadose therapy as per the National Acute Spinal Cord Injury 2 study (NASCI 2) recommendations [30 mg/kg over 30 minutes followed by 5.5 mg/kg/hr over 48 hrs] was followed by oral steroid doses of 1 mg/kg body weight that were tapered over two weeks [10]. The therapy was administered to all patients with visual acuity less than 6/18 who were seen within 24 hours of injury. None of the patients in the study underwent surgical intervention. The presence of an orbital fracture was associated with an increased likelihood of treatment. Six (60%) out of 10 patients with orbital fractures had received treatment and four (40%) patients with orbital fractures did not receive treatment, although this result was not statistically significant (p=0.18).

Visual outcome of steroid treatment

Out of 17 patients that received steroid treatment, six patients (26.08%) showed improvements in three or more lines of VA on Snellen’s visual acuity chart. Two (33.33%) of the six patients in the untreated group showed improvements in three lines of VA on Snellen’s visual acuity chart.

Type of injury and treatment

When the type of injury was analyzed with regards to treatment, the maximum number of treated cases were in the injury with RTA group. The same number of cases was in both the treated and untreated groups in the assault group. In cases that fell, all patients received treatment but this result was not statistically significant when compared to cases that received treatment in the RTA and assault groups (p=0.52).

Presenting visual acuity and treatment

All patients that presented with VA 6/24-6/60 received treatment; 66.7% of patients with VA > 6/18 and 66.7% of patients with VA between 6/60-PL received treatment (p=0.40).

Associated ocular injury and treatment

Analysis of associated ocular injury with treatment revealed that all patients (100%) with posterior segment injury received treatment and four patients (80%) with anterior segment injury received treatment. Eleven patients that did not have ocular injuries received treatment but this result was not statistically significant (p=0.36). All patients with associated anterior and posterior segment injuries had clinical findings that did not account for the visual loss, therefore traumatic optic neuropathy was considered as a diagnosis for these patients.

## Discussion

TON was described by Hippocrates over two thousand years ago and is a cause of severe visual loss after blunt ocular trauma [14]. A rotational or shearing force which is transmitted to the frontal areas in closed-head injuries causes optic nerve damage. The transmitted deformative stress most commonly affects the intracanalicular segment followed by the intracranial portion of the optic nerve. The pathophysiology of TON is thought to be multifactorial, and involves a primary and secondary mechanism of injury. For indirect TON cases, the injury to the axons is due to shearing forces that are transmitted to the fibers or to the vascular supply of the nerve. A secondary mechanism can result in optic nerve swelling following acute injury, which can exacerbate retinal ganglion cell (RGC) degeneration by further compromising the vascular supply, either through a rise in intraluminal pressure or reactive vasospasm.

The incidence of TON following head injuries in various published case series varies between 0.7-2.5% [1,15]. The proportion of traumatic optic neuropathy in relation to head injuries in our study was 1.26% which was in accordance with other studies. All patients in the study were males; no females were included in our study. This is in accordance with various other studies on TON which showed a prevalence in males [16-18] and suggests that males are more prone to trauma compared with females. The median age of the patients in this study was 18 years. This was in contrast to other studies which showed the median age of the patients to be more than 30 years [16-18]. Eleven (47.82%) patients in the study were under 18 years of age which shows that the majority of trauma occurs in the younger age group, and young patients were affected at similar rates to adults. The most common cause of injury in the study was RTA followed by falls and assault. A study by Das et al. reported road traffic injury as the most common cause of injury [19]. Wilhelm reports that the most common etiology of trauma was assault (56%), followed by falls (21%) [15].

The treatment options for TON include observation, systemic steroid therapy, surgical decompression and a combination therapy of steroids and surgery. In our study, 17 patients received intravenous methylprednisolone treatment and six patients did not receive steroid therapy because of neurosurgical contraindications. Steroid therapy has been used to treat TON since the early 1980s. The most common steroid used is methylprednisolone which has multiple mechanisms of action such as arachidonic acid metabolism inhibition [20,21], lipid peroxidation [22], stabilizing lysosomal membranes [23], reducing basal metabolic energy [24], lowering lactate accumulation [25], and enhancing the expression of heat-shock proteins [26]. There is no consensus on the dose and timing of steroid treatment in TON. Studies such as the NASCIS showed the beneficial effects of high-dose steroid treatment and later studies, such as the Cochrane review, have recommended that steroids should not be routinely given in all head injuries. A literature review on steroid doses in TON showed varying steroid regimes which ranged from 100-5400 mg per day. NASCIS II, a multicenter, randomized, double-blind, placebo-controlled trial showed that patients who received steroids within 8 hours of the injury had significantly improved neurological functions as compared to those who were treated after 8 hours. However, adverse survival effects of megadose steroid treatment were found in the CRASH study and various other laboratory research studies have indicated that steroids should not be given routinely in all head injury cases. None of the patients in our study underwent surgery as the systemic conditions did not allow for surgical treatment.

Out of 17 patients that were treated with intravenous methylprednisolone, six patients (35.29%) showed improvement in three lines whereas two (33.33%) out of six patients in the untreated group showed improvement on follow-up; however, this result was not statistically significant. This was in accordance with the study by Lee et al. where 24% of the patients in the treated group and 20% of the patients in the untreated group showed improvement in visual acuity [1]. The presence of an orbital fracture was associated with an increased likelihood of treatment in the study; six out of 10 patients (60%) with orbital fractures received treatment whereas four out of 10 patients (40%) that did not have orbital fractures received treatment. This was in accordance with the study by Lee et al. where 40% of patients with orbital fractures received treatment and 29% of patients without orbital fractures received treatment [18].

The study was conducted in the Indian population where a large number of studies are lacking. The present study highlighted the various demographic factors associated with TON. Associated ocular injuries with TON were also well presented.

The study being retrospective in nature has its major limitation. The study had a small sample size.

## Conclusions

This study showed that there was no significant association between presenting visual acuity and treatment. Neurosurgical contraindications prevented steroid treatment in this study which shows that treatment must be individually tailored. The study showed that the presence of significant ocular and orbital injuries increased the likelihood of treatment.

There was no difference between the steroid-treated and untreated groups with respect to vision improvement. We recommend individually tailored steroid treatment for TON. Additional studies and long-term clinical trials are needed to determine the optimal treatment for TON patients.
